# Oxygen Redox Versus Oxygen Evolution in Aqueous Electrolytes: Critical Influence of Transition Metals

**DOI:** 10.1002/advs.202104907

**Published:** 2022-02-19

**Authors:** Hirohito Umeno, Kosuke Kawai, Daisuke Asakura, Masashi Okubo, Atsuo Yamada

**Affiliations:** ^1^ A Department of Chemical System Engineering School of Engineering The University of Tokyo Hongo 7‐3‐1, Bunkyo‐ku Tokyo 113‐8656 Japan; ^2^ National Institute of Advanced Industrial Science and Technology (AIST) Umezono 1‐1‐1 Tsukuba Ibaraki 305‐8568 Japan

**Keywords:** aqueous batteries, aqueous electrolyte, batteries, cathodes, oxygen evolution, oxygen redox

## Abstract

Aqueous lithium‐ion batteries are promising electrochemical energy storage devices owing to their sustainable nature, low cost, high level of safety, and environmental benignity. The recent development of a high‐salt‐concentration strategy for aqueous electrolytes, which significantly expands their electrochemical potential window, has created attractive opportunities to explore high‐performance electrode materials for aqueous lithium‐ion batteries. This study evaluates the compatibility of large‐capacity oxygen‐redox cathodes with hydrate‐melt electrolytes. Using conventional oxygen‐redox cathode materials (Li_2_RuO_3_, Li_1.2_Ni_0.13_Co_0.13_Mn_0.54_O_2_, and Li_1.2_Ni_0.2_Mn_0.6_O_2_), it is determined that avoiding the use of transition metals with high catalytic activity for the oxygen evolution reaction is the key to ensuring the stable progress of the oxygen redox reaction in concentrated aqueous electrolytes.

## Graphical abstract

By controlling both the water activity of aqueous electrolytes and the catalytic activity of cathode materials, oxygen‐redox reaction is stabilized over oxygen‐evolution reaction, successfully achieving a large capacity of Li‐rich oxides for aqueous batteries.

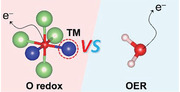

## 1 Introduction

Implementing renewable energy technologies in power grids is imperative for ensuring sustainability. Clean electricity from renewable energy resources can help mitigate climate change and prevent the environmental pollution arising from fossil fuel combustion. However, as renewable energy technologies involve natural processes (e.g., sunlight and wind), their power supply is intrinsically intermittent, and this severely degrades the quality of the electricity supply.^[^ ^1^ ^]^ Therefore, the load leveling of the intermittent power from renewable energy sources is important.

The use of large‐scale electrochemical energy storage devices is an attractive option for ensuring the flexibility of the power grid, which, in turn, would guarantee an efficient and steady power supply. However, lithium‐ion batteries, which power most portable electronic devices, are not suitable for this purpose because of their high fabrication/maintenance costs and limited calendar life.^[^ ^2^, ^3^ ^]^ In particular, flammable organic electrolytes pose an unacceptable fire accident risk while requiring rigorous manufacturing conditions to avoid water contamination.^[^ ^4^ ^]^ Consequently, lithium‐ion batteries based on aqueous electrolytes are an important technical option for large‐scale applications, offsetting both the high fabrication/maintenance costs and fire accident risk of their flammable counterparts.^[^ ^5^, ^6^, ^7^ ^]^

Even though significant efforts have been devoted to the development of aqueous lithium‐ion batteries for decades, a major obstacle has been their low operational voltage owing to the intrinsically narrow electrochemical potential window of water. The low operational voltage results in a small energy density and severely limits the options available for both positive and negative electrodes. However, a high‐salt‐concentration strategy developed by Xu et al.^[^ ^8^ ^]^ and Yamada et al.^[^ ^9^ ^]^ (involving the use of so‐called water‐in‐salt or hydrate‐melt electrolytes) can expand the electrochemical potential window of aqueous electrolytes to more than 3 V. This allows for the use of a greater range of electrodes and should lead to the development of high‐performance electrode materials compatible with highly concentrated aqueous electrolytes.^[^ ^10^, ^11^, ^12^, ^13^, ^14^, ^15^, ^16^, ^17^ ^]^

In this work, we evaluate the compatibility of lithium‐rich transition‐metal oxides Li_1+_ *_x_* M_1‐_ *_x_* O_2_ (M: transition metal) with an aqueous electrolyte. A large capacity (>200 mAh g^‐1^) accumulating both M‐ and oxygen‐redox reactions,^[^ ^18^, ^19^, ^20^ ^]^ is essential for increasing the energy density of aqueous batteries. However, the oxygen redox activity possible using highly concentrated aqueous electrolytes has rarely been investigated.^[^ ^21^ ^]^ Herein, we report the oxygen redox activities of three oxygen‐redox positive electrodes (Li_2_RuO_3_, Li_1.2_Ni_0.13_Co_0.13_Mn_0.54_O_2_, and Li_1.2_Ni_0.2_Mn_0.6_O_2_) and elucidate the critical influence of transition metals on oxygen redox activity in aqueous systems.

## 2 Results and Discussion

Li_1.2_Ni_0.13_Co_0.13_Mn_0.54_O_2_ and Li_1.2_Ni_0.2_Mn_0.6_O_2_ were synthesized by sintering their co‐precipitated precursors. Li_2_RuO_3_ was synthesized by a solid‐state method. The X‐ray diffraction patterns of all the samples are consistent with those reported previously,^[^ ^22^, ^23^, ^24^, ^25^ ^]^ thus confirming the successful synthesis of the lithium‐rich oxides (Figure S1, Supporting Information). These lithium‐rich oxides exhibit capacities greater than 230 mAh g^‐1^ in an organic electrolyte (1 м LiPF_6_ in ethylene carbonate (EC)/dimethyl carbonate (DMC) = 1:1(vol.)), as is typical for oxygen redox positive electrode materials (Figure S2, Supporting Information).^[^ ^22^, ^23^, ^24^, ^25^ ^]^

**Figure 1:**
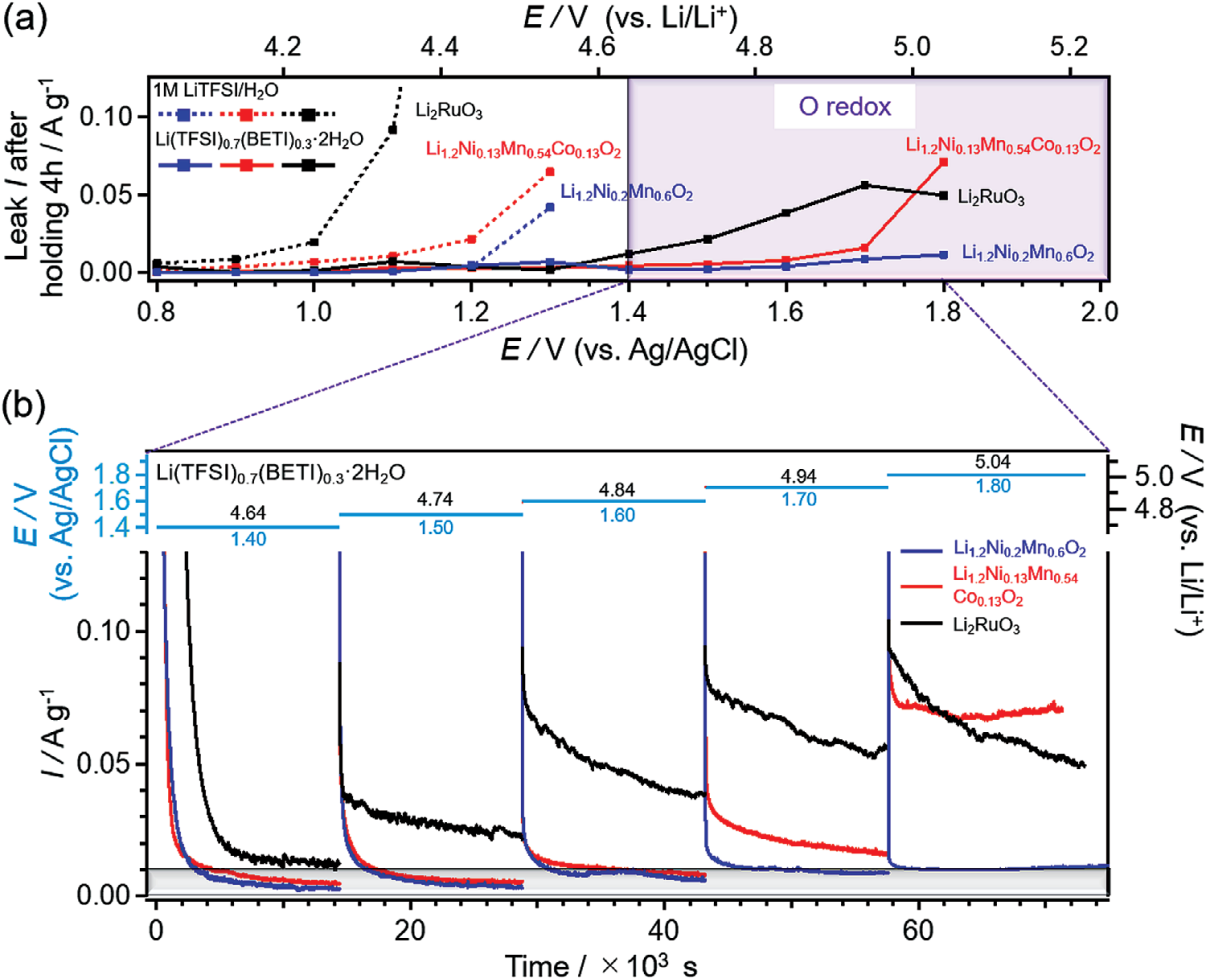
a) Leakage current values after holding Li_1.2_Ni_0.2_Mn_0.6_O_2_ (blue), Li_1.2_Ni_0.13_Mn_0.54_Co_0.13_O_2_ (red), and Li_2_RuO_3_ (black) electrodes at constant potential in Li(TFSI)_0.7_(BETI)_0.3_·2H_2_O (solid line) and 1 м LiTFSI aqueous solution (dotted line) for 4 h. b) Chronoamperograms of Li_1.2_Ni_0.2_Mn_0.6_O_2_ (blue), Li_1.2_Ni_0.13_Co_0.13_Mn_0.54_O_2_ (red), and Li_2_RuO_3_ (black) electrodes in Li(TFSI)_0.7_(BETI)_0.3_·2H_2_O electrolyte.

To evaluate the compatibility of the oxygen‐redox electrodes with dilute and concentrated aqueous electrolytes, chronoamperometry was performed using these electrolytes at different potentials in the float mode. The time‐dependent anodic‐current responses in a 1 м lithium bis(trifluoromethanesulfonyl)imide (LiTFSI) aqueous electrolyte are shown in Figure S3 (Supporting Information). After the application of a constant potential for 4 h, each electrode approaches the steady state and exhibits an anodic leakage current arising from continuous side reactions. **Figure** **1**a shows the steady‐state anodic leak current as a function of the applied potential for Li_2_RuO_3_, Li_1.2_Ni_0.13_Co_0.13_Mn_0.54_O_2_, and Li_1.2_Ni_0.2_Mn_0.6_O_2_. Of the three materials, Li_2_RuO_3_ induced an anodic leakage current at the lowest onset potential, which is greater than 1.0 V versus Ag/AgCl (4.14 V vs Li/Li^+^); the anodic leakage current exceeds 20 mA g^‐1^ presumably owing to continuous electrolyte oxidation. Although Li_1.2_Ni_0.13_Co_0.13_Mn_0.54_O_2_ and Li_1.2_Ni_0.2_Mn_0.6_O_2_ show slightly higher onset potentials of 1.1 and 1.2 V versus Ag/AgCl, respectively, these potential limits are too low to trigger their oxygen‐redox activities. Indeed, charging Li_2_RuO_3_ in a 1 м LiTFSI aqueous electrolyte to 0.9 V versus Ag/AgCl results in a small capacity of 119 mAh g^‐1^ while causing continuous electrolyte oxidation at voltages greater than 0.9 V versus Ag/AgCl (Figure S4a, Supporting Information). Similarly, neither Li_1.2_Ni_0.13_Co_0.13_Mn_0.54_O_2_ nor Li_1.2_Ni_0.2_Mn_0.6_O_2_ shows a large reversible capacity in the 1 м LiTFSI aqueous electrolyte. Therefore, the oxygen redox activities of these materials cannot be exploited using the 1 м LiTFSI aqueous electrolyte owing to the narrow electrochemical potential window.

On the other hand, a high‐salt‐concentration strategy can trigger oxygen redox activity. The onset potential of the anodic leakage current of Li_2_RuO_3_ is raised to 1.4 V versus Ag/AgCl (4.64 V vs Li/Li^+^) in the hydrate‐melt electrolyte Li(TFSI)_0.7_(BETI)_0.3_·2H_2_O (Figure 1b). Importantly, the suppression of the anodic side reactions aids the activation of oxygen redox reactions, resulting in a capacity as large as ≈300 mAh g^‐1^ (Figure S4b, Supporting Information). However, during repeated charge/discharge cycling, the reversible capacity of Li_2_RuO_3_ decreases steadily, presumably because of damaging parasitic reactions such as the Li^+^‐H^+^ exchange,^[^ ^26^ ^]^ or the oxygen evolution reaction (OER).^[^ ^27^ ^]^

**Figure 2:**
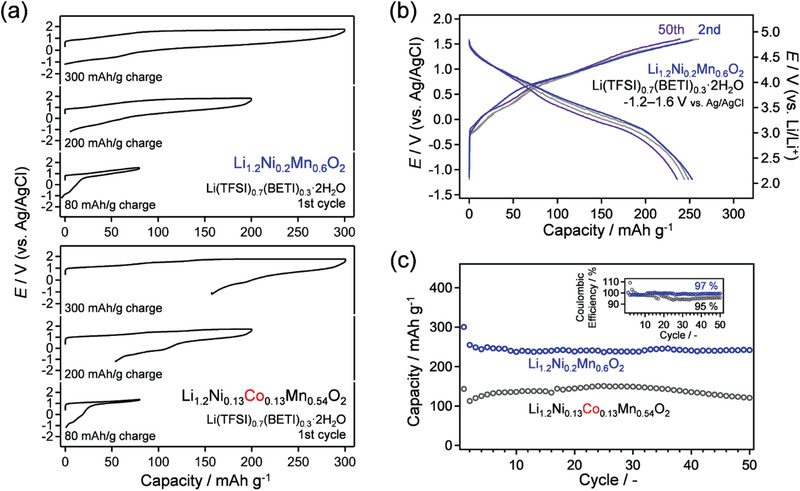
a) First charge–discharge cycle curves of Li_1.2_Ni_0.2_Mn_0.6_O_2_ and Li_1.2_Ni_0.13_Co_0.13_Mn_0.54_O_2_ in Li(TFSI)_0.7_(BETI)_0.3_·2H_2_O under limit of charge capacity (80, 200, and 300 mAh g^‐1^). Specific current is 50 mA g^‐1^. b) Charge–discharge curves of Li_1.2_Ni_0.2_Mn_0.6_O_2_ upon cycling in Li(TFSI)_0.7_(BETI)_0.3_·2H_2_O with cutoff voltage ranging from −1.2 to 1.6 V versus Ag/AgCl. c) Capacity retention and Coulombic efficiencies of Li_1.2_Ni_0.2_Mn_0.6_O_2_ (blue squares) and Li_1.2_Ni_0.13_Co_0.13_Mn_0.54_O_2_ (black squares) in a Li(TFSI)_0.7_(BETI)_0.3_·2H_2_O electrolyte.

The onset potential is further up‐shifted to 1.7 and 1.8 V versus Ag/AgCl using Li_1.2_Ni_0.13_Co_0.13_Mn_0.54_O_2_ and Li_1.2_Ni_0.2_Mn_0.6_O_2_, respectively, in a hydrate‐melt electrolyte (Figure 1a,b). These voltage limits allow for the realization of a charge capacity of 300 mAh g^‐1^ for both electrodes during the first charge (**Figure** **2**a). However, the first discharge capacity of cobalt‐containing Li_1.2_Ni_0.13_Co_0.13_Mn_0.54_O_2_ is 143 mAh g^‐1^ with a Coulombic efficiency of 48%, suggesting the simultaneous occurrence of competing anodic side reactions such as electrolyte oxidation. In contrast, cobalt‐free Li_1.2_Ni_0.2_Mn_0.6_O_2_ delivers a discharge capacity of 298 mAh g^‐1^ at the first discharge with a Coulombic efficiency of ≈100%, which is much higher than that with an organic electrolyte (78%) (Figure S2, Supporting Information). A possible explanation for the additional discharge capacity at the first cycle is the reduction of O_2_ gas dissolved in the electrolyte, because O_2_ gas evolution from the cathode surface occurs during the first charge process.^[^ ^28^ ^]^ Alternatively, or in parallel, as the highly concentrated aqueous electrolytes have the higher electrochemical stability against anodic oxidation than conventional organic electrolytes,^[^ ^9^ ^]^ parasitic irreversible reactions during the first^s^ charge such as electrolyte decomposition and cation densification are suppressed to provide the higher initial Coulombic efficiency. The voltage decay upon cycling is mitigated relative to that for the organic electrolyte (0.06 V decay after 50 cycles for the aqueous electrolyte while 0.11 V decay for the organic electrolyte), because the capacity with the aqueous electrolyte is smaller than that with the organic electrolyte. Importantly, 81% of the initial discharge capacity is retained after 50 charge–discharge cycles (Figure 2b,c), indicating that the operation of the oxygen redox electrode in the concentrated aqueous electrolyte is stable when ruthenium and cobalt are not present in the cathode material.

**Figure 3:**
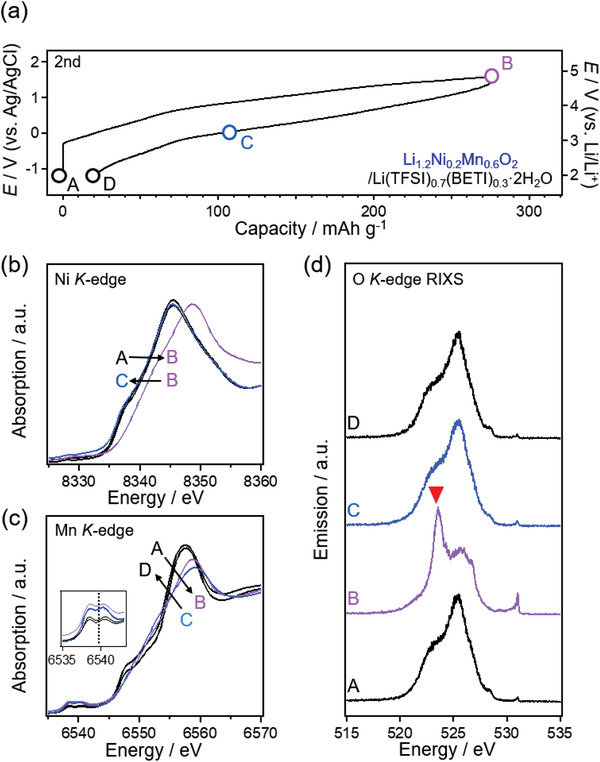
a) Charge–discharge curve of Li_1.2_Ni_0.2_Mn_0.6_O_2_ during the second cycle in Li(TFSI)_0.7_(BETI)_0.3_·2H_2_O. Changes in b) Ni, and c) Mn *K*‐edge X‐ray absorption spectra. d) Evolution of O *K*‐edge resonant inelastic X‐ray scattering (RIXS) spectra excited using the incident photon energy of 531.0 eV.

To confirm the occurrence of the oxygen redox reaction in Li_1.2_Ni_0.2_Mn_0.6_O_2_, ex situ X‐ray absorption–emission spectroscopy was conducted during the second cycle (**Figure** **3**a–d). A reversible peak shift is observed in the low‐voltage and high‐voltage regions for the Mn and Ni *K*‐edges, respectively (Figure 3b,c), highlighting the contributions of both Mn and Ni to the cationic redox capacity. The O *K*‐edge X‐ray absorption spectra show that the characteristic absorption at 531 eV emerges/diminishes reversibly during charging/discharging (Figure S5, Supporting Information). Furthermore, the O *K*‐edge resonant inelastic X‐ray scattering (RIXS) spectra obtained using an incident photon of 531 eV show a sharp X‐ray emission at 523 eV after charging, which is widely recognized as the fingerprint of oxygen oxidation (Figure 3d). All these spectroscopic features are consistent with those reported previously for oxygen redox electrodes in organic electrolytes.^[^ ^28^, ^29^, ^30^, ^31^, ^32^ ^]^

**Figure 4:**
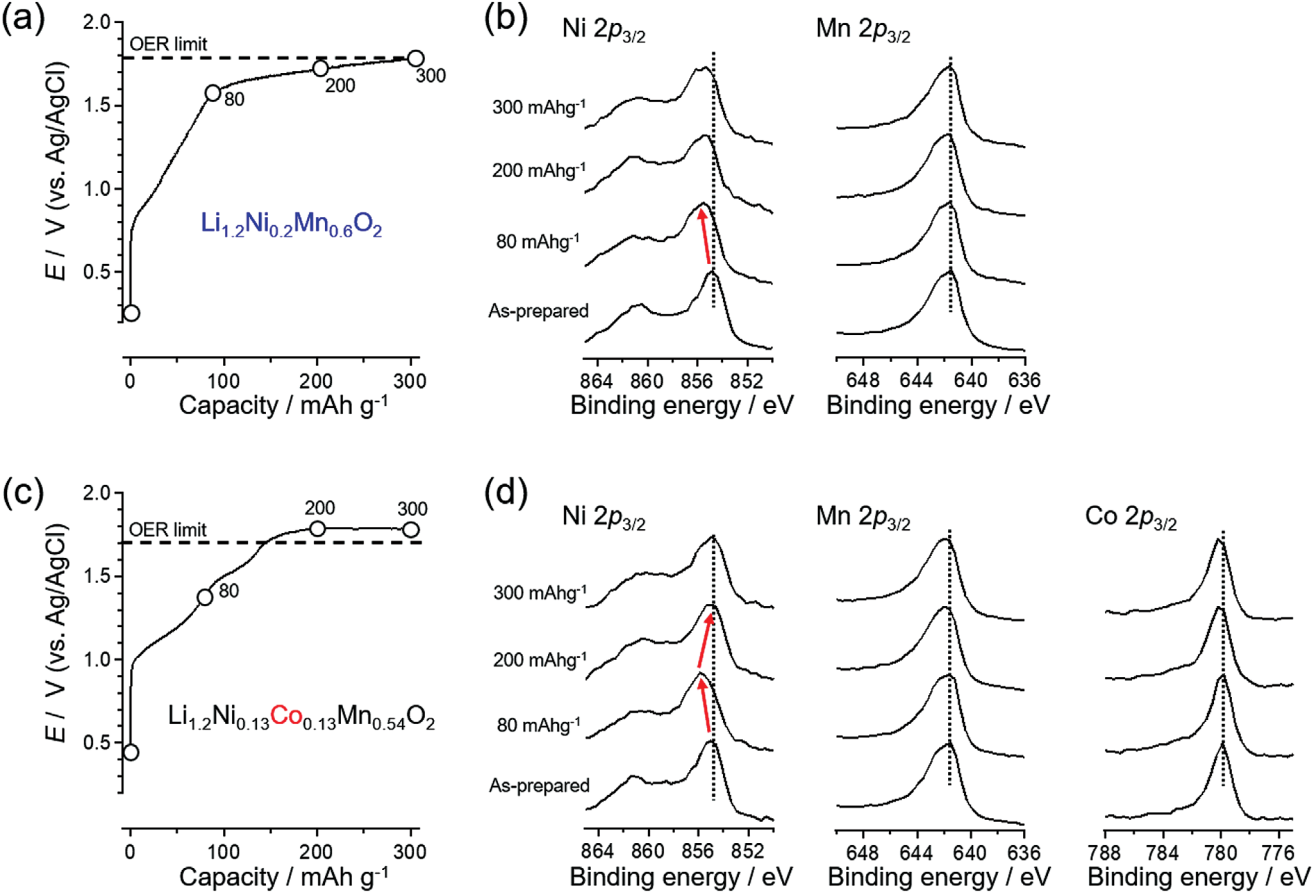
a) Potential profile and b) XPS spectra of Li_1.2_Ni_0.2_Mn_0.6_O_2_, and c) potential profile and d) XPS spectra of Li_1.2_Ni_0.13_Co_0.13_Mn_0.54_O_2_ during the first charge at 50 mA g^‐1^ in Li(TFSI)_0.7_(BETI)_0.3_·2H_2_O.

An important question to ask here is why only cobalt‐free Li_1.2_Ni_0.2_Mn_0.6_O_2_ exhibits a reversible oxygen redox capacity in the hydrate‐melt system. To determine how cobalt disables the oxygen redox activity, X‐ray photoelectron spectroscopy (XPS) was conducted during the first charge in order to evaluate the difference in the surface states. In the case of Li_1.2_Ni_0.2_Mn_0.6_O_2_, the peak at a binding energy of 854.5 eV, which is related to the Ni 2*p*_3/2_ core, blue‐shifts to 856 eV after charging to 80 mAh g^‐1^ (≈ 1.6 V vs Ag/AgCl). This is followed by the emergence of a new higher‐energy shoulder at 857 eV after charging to 300 mAh g^‐1^ (≈1.8 V vs Ag/AgCl; **Figure** **4**a,b), indicating the continuous oxidation of nickel at the surface. The XPS peak for the Mn 2*p*_3/2_ core does not change during the first charge because Mn^4+^ in the pristine state cannot be oxidized, confirming reasonable surface oxidation in association with the first charge process.

In striking contrast, when Co‐containing Li_1.2_Ni_0.13_Co_0.13_Mn_0.54_O_2_ is charged to 80 mAh g^‐1^, the XPS peak at 854.5 eV, which is related to the Ni 2*p*_3/2_ core, shifts to 856 eV. However, it returns to 854.5 eV upon further charging to 200 and 300 mAh g^‐1^ (Figure 4c,d), indicating Ni undergoes reduction on the particle surfaces owing to the exposure to the large‐overpotential environment. It is noteworthy that the voltage plateau at ≈1.5 V versus Ag/AgCl, which is characteristic of the initial activation process of the oxygen‐redox electrodes, is less obvious than that of Li_1.2_Ni_0.2_Mn_0.6_O_2_. Presumably, the oxidized oxide ion in Li_1.2_Ni_0.13_Co_0.13_Mn_0.54_O_2_ is unstable in aqueous electrolytes to disrupt this activation process.

**Scheme 1:**
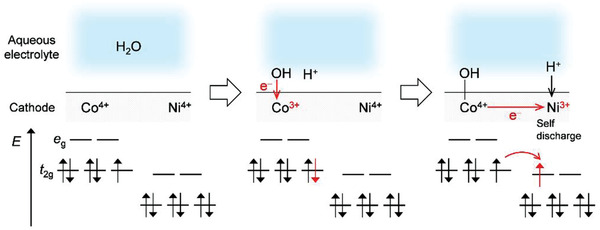
Schematic illustration of self‐discharge process in Li_1.2_Ni_0.13_Co_0.13_Mn_0.54_O_2_.

The reduction of Ni at the surface of Li_1.2_Ni_0.13_Co_0.13_Mn_0.54_O_2_ is attributable to the high catalytic activity of Co with respect to the oxygen evolution reaction (OER). Layered oxides containing Co, such as CoOOH,^[^ ^33^ ^]^ Li_1‐_ *_x_* CoO_2_,^[^ ^34^, ^35^ ^]^ and Na_0.67_CoO_2_,^[^ ^36^ ^]^ have been reported to exhibit high OER activity. Consistently, Li_1.2_Ni_0.13_Co_0.13_Mn_0.54_O_2_ exhibits a slightly larger anodic leakage current in the high‐voltage region than that of cobalt‐free Li_1.2_Ni_0.2_Mn_0.6_O_2_, as shown in Figure 1b. Presumably, the high OER‐activity of cobalt at the surface accelerates the OER, 2H_2_O → O_2_ + 4H^+^ + 4e^‒^, which is initiated by the primary anodic process of Co^4+^ + H_2_O → Co^3+^‐OH + H^+^.^[^ ^37^, ^38^ ^]^ However, as the electrochemical potential of the electrons of Ni^4+^ in charged Li_1.2_Ni_0.13_Co_0.13_Mn_0.54_O_2_ is lower than that of Co^3+^, the spontaneous reduction of Ni occurs as follows: Co^4+^ + Ni^4+^ + H_2_O → Co^3+^‐OH + Ni^4+^ + H^+^ → Co^4+^‐OH + Ni^3+^ + H^+^ (**Scheme** **1**). This self‐discharging process arising from the parasitic side reaction inhibits the oxygen redox activity of Li_1.2_Ni_0.13_Co_0.13_Mn_0.54_O_2_. It is also important to note that a Li_1‒_ *_x_* CoO_2_ electrode stably operates with a Co^4+/3+^ redox couple in highly concentrated electrolytes.^[^ ^9^ ^]^ However, the charged Li_1.2_Ni_0.13_Co_0.13_Mn_0.54_O_2_ electrode possesses an electronic state consisting of Co^4+^ and oxidized oxide ions such as O^‒^ and/or O_2_ *^n^* ^‒^.^[^ ^39^ ^]^ As the oxidation of oxide ion causes the M‒O bond dissociation, the resulting Co in an undercoordination environment should be highly electrophilic, leading to the self‐discharge process in Scheme 1. Therefore, in addition to employing the high‐salt‐concentration strategy for expanding the electrochemical window, it is also essential to avoid using the OER‐active transition metals in large‐capacity oxygen‐redox cathodes in aqueous media. As demonstrated in this study, highly OER‐active Ru and Co cannot be considered as the primary options when designing aqueous oxygen‐redox batteries.

**Figure 5:**
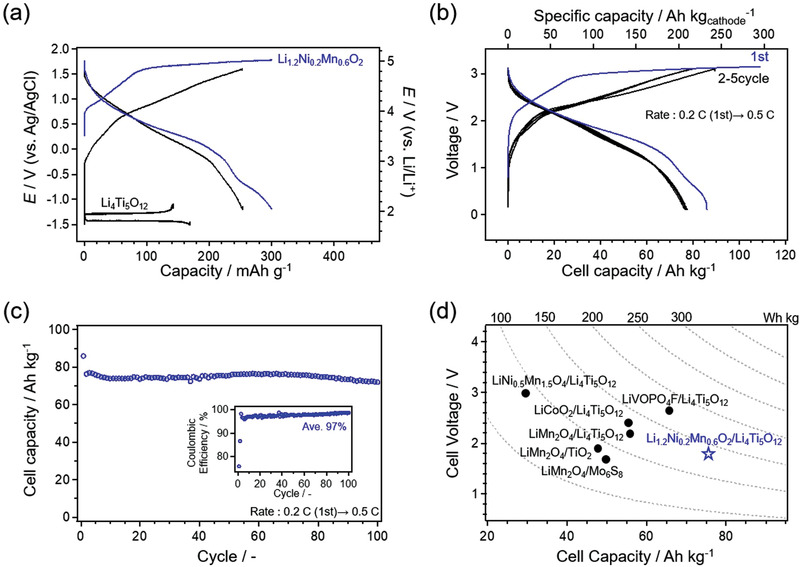
a) Charge–discharge curves of Li_1.2_Ni_0.2_Mn_0.6_O_2_ and Li_4_Ti_5_O_12_ in Li(TFSI)_0.7_(BETI)_0.3_·2H_2_O electrolyte. b) Voltage profile of Li_1.2_Ni_0.2_Mn_0.6_O_2_‐Li_4_Ti_5_O_12_ full cell in Li(TFSI)_0.7_(BETI)_0.3_·2H_2_O electrolyte at a specific current of 125 mA g^‐1^ after the first cycle at 0.2 C. Cell capacity is normalized based on total weight of active materials. c) Cycling stability and Coulombic efficiency of full cell. d) Comparison of performances of aqueous lithium‐ion batteries with highly concentrated aqueous electrolytes.

To further confirm the stable operation of the oxygen redox cathode in the hydrate‐melt system, we fabricated a full cell with the Li_1.2_Ni_0.2_Mn_0.6_O_2_| Li(TFSI)_0.7_(BETI)_0.3_·2H_2_O|Li_4_Ti_5_O_12_ configuration (**Figure** **5**a). After the first activation cycle, the full cell exhibits a specific capacity of 82 Ah per unit weight (kg) of the active materials (224 Ah per unit weight (kg) of the cathode material) at a rate of 0.5 C with an average discharge voltage of 1.75 V and the energy efficiency of 66% (Figure 5b). In other words, it shows an energy density equivalent to or higher than those of the state‐of‐the‐art aqueous lithium‐ion batteries (Figure 5d).^[^ ^8^, ^9^, ^40^, ^41^, ^42^, ^43^ ^]^ 81% and 48% of the capacity at 0.5 C rate are retained at the rates of 1 C and 2 C, respectively, supporting the stable operation of the system at high rates (Figure S6, Supporting Information). Notably, 87% of the initial cell capacity is retained after 100 cycles while the average Coulombic efficiency is 97% over 100 cycles (Figure 5c). The full‐cell capacity was designed to be limited by the anode capacity (Figure 5a); thus, it is possible to further increase the energy density by balancing the anode/cathode capacities.

## 3 Conclusion

We demonstrated that additional capacity related to the oxygen redox reaction can be activated in a lithium‐rich layered cathode using a highly concentrated aqueous electrolyte. While extending the voltage window using the high‐salt‐concentration strategy is essential for “activating” the oxygen redox reaction, the suppression of the competitive OER is the key to “stabilizing” the reaction, and ensuring long‐term repeated cycling. The presence of highly OER‐active transition metals, such as ruthenium and cobalt, significantly accelerates the OER and the subsequent parasitic side reactions, causing rapid degradation. To further demonstrate the stable operation of an aqueous lithium‐ion battery that utilizes the extra capacity of the oxygen redox reaction in the cathode material, a full cell with the Li_1.2_Ni_0.2_Mn_0.6_O_2_| Li(TFSI)_0.7_(BETI)_0.3_·2H_2_O|Li_4_Ti_5_O_12_ configuration was fabricated. The cell showed reasonable long‐term stability as well as a record‐high energy density. This work highlights the importance of employing an integrated design approach for the electrodes and electrolytes for high‐energy‐density aqueous batteries based on the oxygen redox.

## 4 Experimental Section

Li_1.2_Ni_0.2_Mn_0.6_O_2_ and Li_1.2_Ni_0.13_Co_0.13_Mn_0.54_O_2_ were synthesized via a co‐precipitation method. Nickel sulfate hexahydrate (NiSO_4_·6H_2_O), manganese sulfate pentahydrate (MnSO_4_·5H_2_O), cobalt sulfate heptahydrate (CoSO_4_·7H_2_O), and sodium carbonate (Na_2_CO_3_) were used as the starting materials to prepare the Ni*_x_* Co*_y_* Mn*_z_* CO_3_ precursors. The precursors were thoroughly mixed with Li_2_CO_3_ (5% excess Li) by ball milling and then calcined at 900 °C for 12 h after being pre‐sintered at 500 °C for 6 h. Li_2_RuO_3_ was synthesized using a solid‐state method. Stoichiometric amounts of RuO_2_ and Li_2_CO_3_ (10% excess, to compensate for its volatilization at high temperatures) were thoroughly mixed by ball milling and then calcined at 1000 °C for 24 h.

LiTFSI (LiN(SO_2_CF_3_)_2_) and LiBETI (LiN(SO_2_C_2_F_5_)_2_) were purchased from Kishida Chemical. The hydrate‐melt electrolyte, Li(TFSI)_0.7_(BETI)_0.3_·2H_2_O, was prepared by dissolving LiTFSI and LiBETI in ultrapure water in a molar ratio of LiTFSI/LiBETI/H_2_O = 7:3:20. Li_4_Ti_5_O_12_ was obtained from Ishihara Sangyo. To prepare the electrode films (of Li_1.2_Ni_0.2_Mn_0.6_O_2_ and Li_4_Ti_5_O_12_), the active material powder was mixed with acetylene black (AB, HS‐100, Denki Kagaku Kogyo) and polyvinylidene difluoride (PVDF, Kureha) in *N*‐methylpyrrolidone (NMP, Wako) as solvent; the active material/AB/PVDF weight ratios used were 80:10:10 and 85:10:5 for Li_1.2_Ni_0.2_Mn_0.6_O_2_ and Li_4_Ti_5_O_12_, respectively. The obtained slurry was uniformly spread onto a current collector using a doctor's blade and dried at 60 °C under vacuum overnight. The current collector used for the negative electrodes (Li_4_Ti_5_O_12_) was Al foil while that used for the positive electrodes (Li_1.2_Ni_0.2_Mn_0.6_O_2_) was Ti foil. The galvanostatic curves were recorded using a TOSCAT‐3100 battery tester.

All the electrochemical measurements, except for the charge/discharge tests, were performed using a VMP potentiostat (BioLogic). Cyclic voltammetry measurements were performed using a three‐electrode cell that contained an excess amount of the counter electrode (Pt, Li_x_FePO_4_, active carbon) compared with that of the working electrode. In addition, it used a Ag/AgCl (in a saturated KCl aqueous solution) reference electrode. The scan rate used was 0.1 mV s^–1^. The electrochemical stability windows of the electrolyte solutions were determined via linear sweep voltammetry performed at a scan rate of 0.1 mV s^–1^ using a three‐electrode cell. Full cell tests were performed with a TOSCAT‐3100 charge/discharge unit (Toyo System), using a 2032‐type coin cell with a Li_4_Ti_5_O_12_ negative electrode, Li_1.2_Ni_0.2_Mn_0.6_O_2_ positive electrode, and glass fiber separator. For the Li_1.2_Ni_0.2_Mn_0.6_O_2_/Li_4_Ti_5_O_12_ cells, the negative/positive capacity ratio was set to ≈1.3, based on the theoretical capacities of 230 mAh g^–1^ for Li_1.2_Ni_0.2_Mn_0.6_O_2_ and 155 mAh g^–1^ for Li_4_Ti_5_O_12_. All electrochemical measurements were performed at 25 ℃.

The X‐ray absorption spectroscopy (XAS) measurements were performed at BL‐9C (for the Ni and Mn *K*‐edge) of the Photon Factory, KEK, Japan, and BL07LSU (for the O *K*‐edge) of SPring‐8, Japan. The transmission mode was used for the Mn and Ni *K*‐edge XAS measurements, while the partial‐fluorescence‐yield detection mode was used for the O *K*‐edge XAS measurements. The RIXS measurements were performed using the high‐resolution X‐ray emission spectrometer HORNET at BL07LSU of SPring‐8.^[^ ^44^ ^]^ The total energy resolution of the RIXS measurements was 100 meV at 530 eV. The samples were sealed in a water‐resistant polymer film in an Ar‐filled glove box. For both the XAS and RIXS measurements performed at BL07LSU, the samples were transferred from an Ar‐filled glove box to a vacuum chamber without exposure to air.

The XPS measurements were performed using an X‐ray photoelectron spectrometer (OHI5000 VersaProbe II, ULVAC‐PHI) with a monochromatic Al *Kα* X‐ray source. A three‐electrode cell was subjected to charge/discharge measurements at 0.2 C and then disassembled in an Ar‐filled glove box. Subsequently, the cycled working electrode (Li_1.2_Ni_0.2_Mn_0.6_O_2_, Li_1.2_Ni_0.13_Co_0.13_Mn_0.54_O_2_) was washed thrice with 1,2‐dimethoxyethane to minimize the amount of residual Li salts. The washed electrode was then dried and transferred to the XPS chamber without exposure to air.

## Conflict of Interest

The authors declare no conflict of interest.

## Supporting information

Supporting Information

## Data Availability

The data that support the findings of this study are available from the corresponding author upon reasonable request.
