# Supplementary material for: Oxygen Redox Versus Oxygen Evolution in Aqueous Electrolytes: Critical Influence of Transition Metals
Source: Adv Sci (Weinh). 2022 Feb 19;9(12):2104907. doi: 10.1002/advs.202104907 (PMC9035997; doi:10.1002/advs.202104907)
Supplement: Supplementary file 1 — Supporting Information [file ADVS-9-2104907-s001.pdf]

WILEY-VCH

ADVANCED SCIENCE
Open Access

Supporting Information

for *Adv. Sci.*, DOI: 10.1002/advs.202104907

Oxygen redox versus oxygen evolution in aqueous electrolytes: critical influence of transition metals

*Hirohito Umeno, Kosuke Kawai, Daisuke Asakura, Masashi Okubo, and Atsuo Yamada**

Supporting Information

Oxygen redox versus oxygen evolution in aqueous electrolytes: critical influence of transition metals

*Hirohito Umeno, Kosuke Kawai, Daisuke Asakura, Masashi Okubo, and Atsuo Yamada**

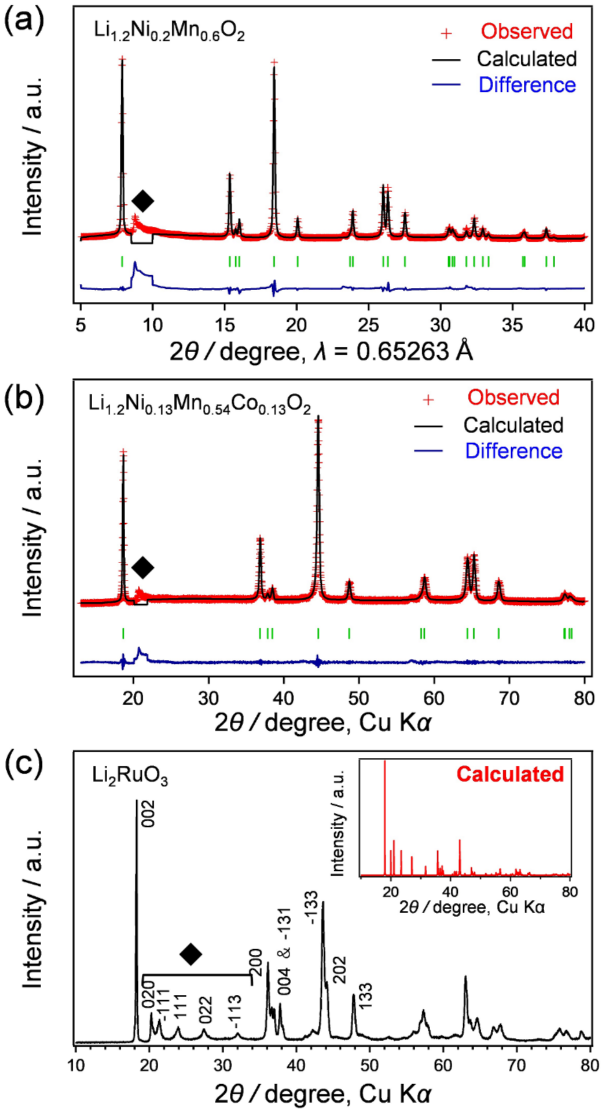

Figure S1. XRD patterns and Rietveld refinement results of (a) $Li_{1.2}Ni_{0.2}Mn_{0.6}O_2$ and (b) $Li_{1.2}Ni_{0.13}Mn_{0.54}Co_{0.13}O_2$. (c) XRD patterns of Li_2RuO_3. ◆ marks show the typical [$\sqrt{3} \times \sqrt{3}$] superstructure in layered oxides.

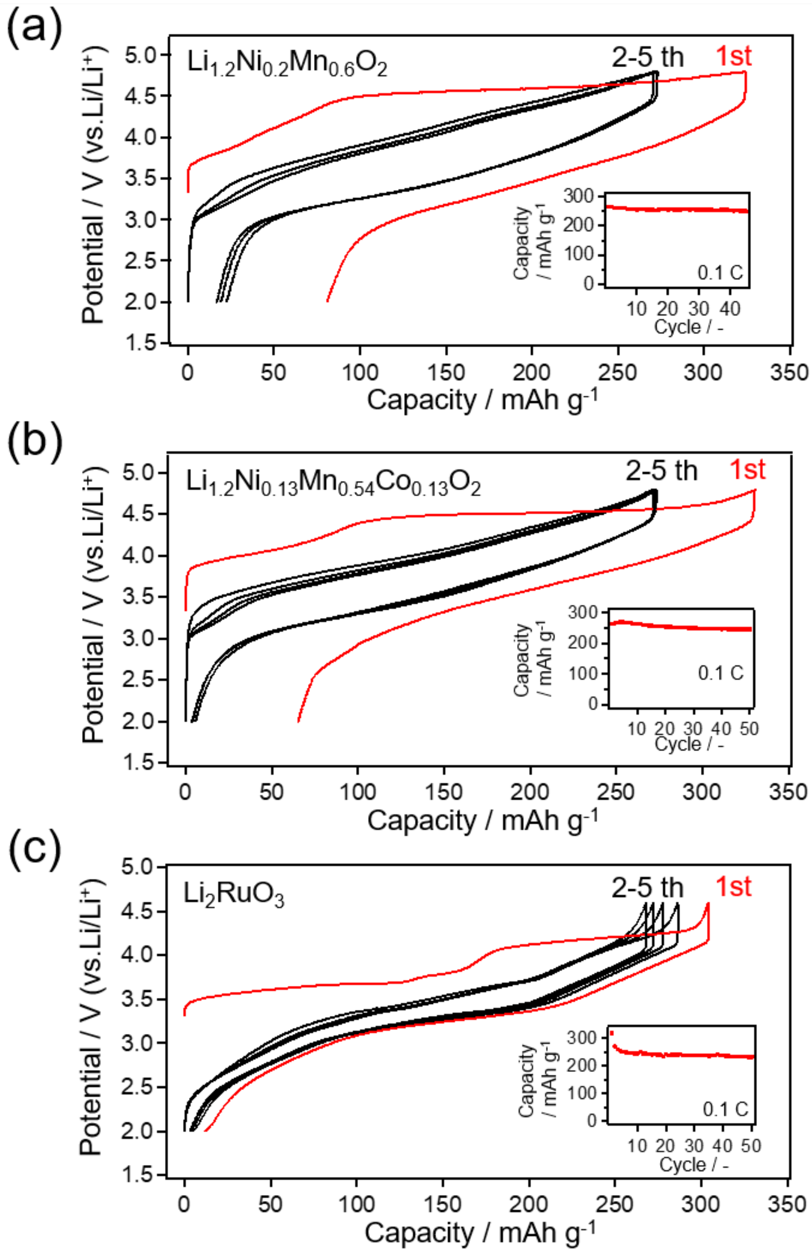

Figure S2. Galvanostatic charge/discharge curves of (a) $Li_{1.2}Ni_{0.2}Mn_{0.6}O_2$, (b) $Li_{1.2}Ni_{0.13}Mn_{0.54}Co_{0.13}O_2$, and (c) Li_2RuO_3 at 0.1 C (1C = 250 mA g^{-1}) in an organic electrolyte (1 M $LiPF_6$ in EC : DMC = 1:1(vol.)).

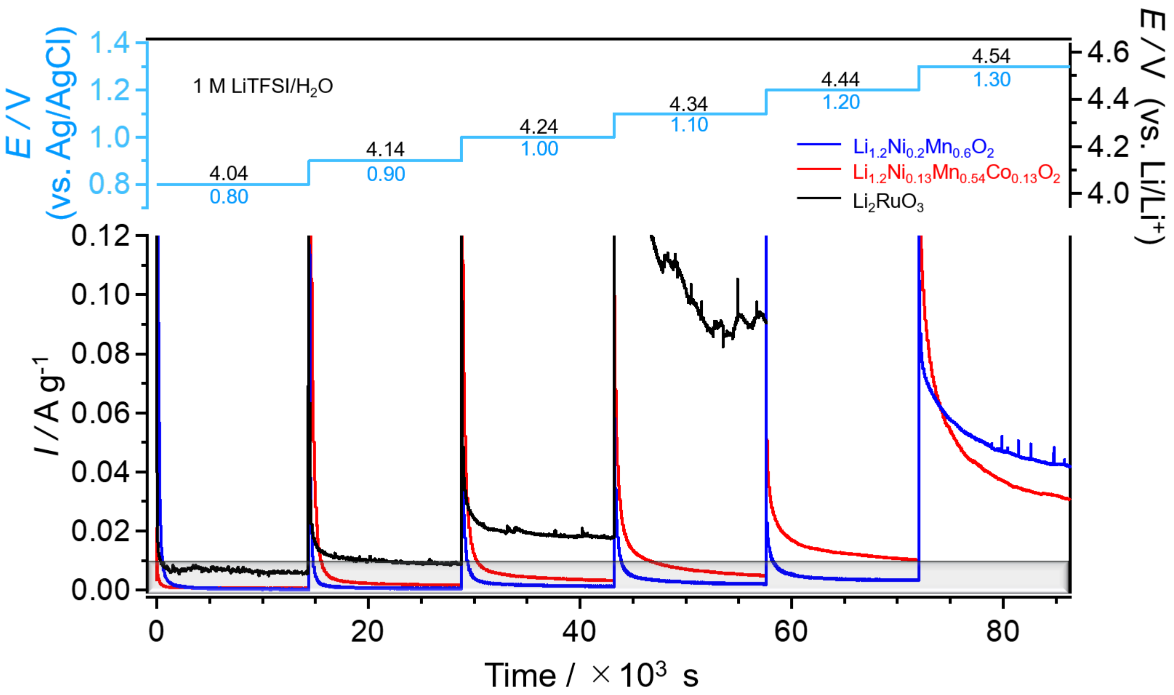

Figure S3. Chronoamperogram of $Li_{1.2}Ni_{0.2}Mn_{0.6}O_2$ (blue), $Li_{1.2}Ni_{0.13}Co_{0.13}Mn_{0.54}O_2$ (red), and Li_2RuO_3 (black) electrodes in a 1 M LiTFSI/H_2O electrolyte.

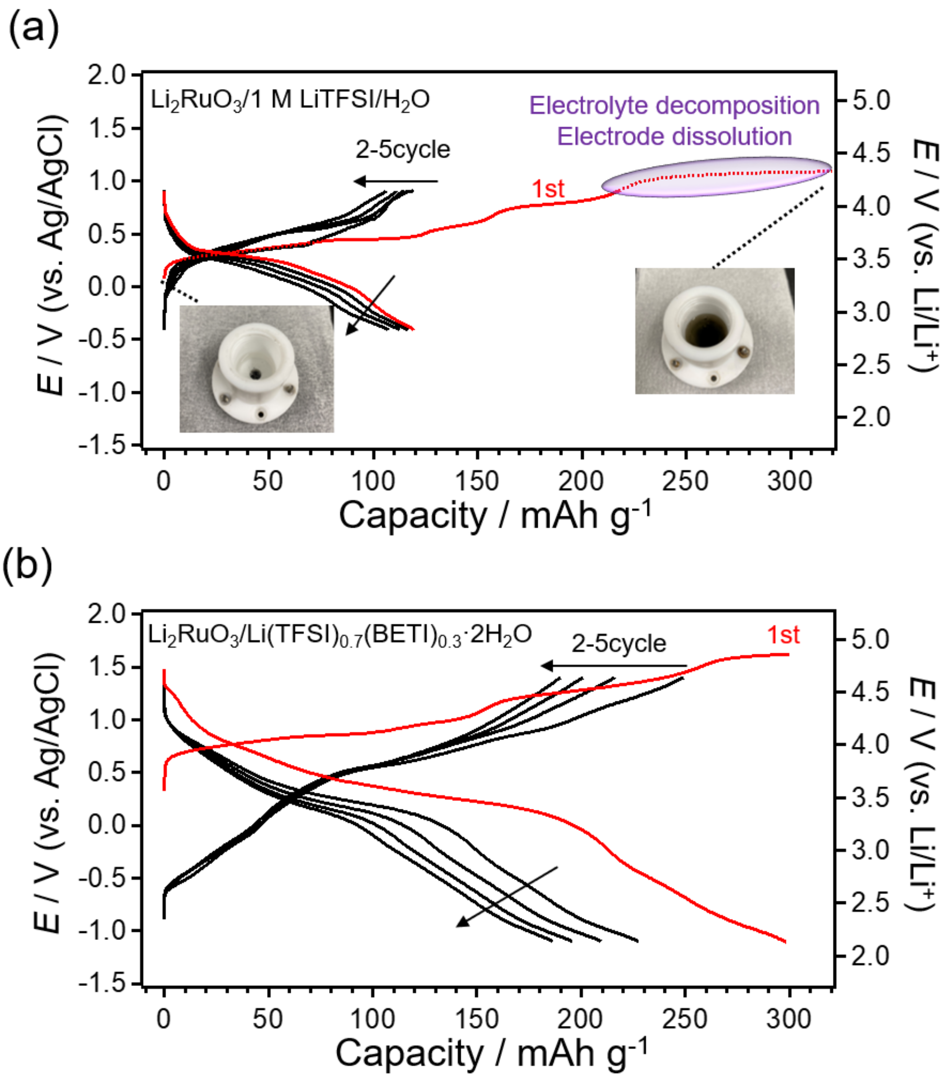

Figure S4. Galvanostatic charge/discharge curves of Li_2RuO_3 at 0.2 C (1C = 250 mA g^{-1}) in (a) 1 M LiTFSI/H_2O and (b) a hydrate-melt electrolyte.

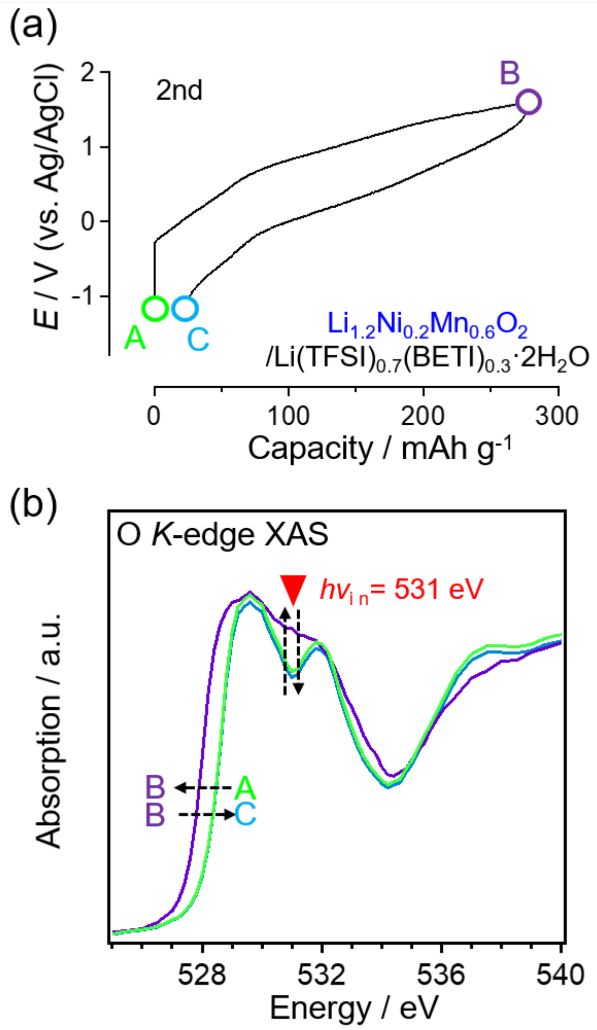

Figure S5. (a) Galvanostatic charge/discharge curves, and (b) *ex situ* O *K*-edge X-ray absorption spectra of $Li_{1.2}Ni_{0.2}Mn_{0.6}O_2$ at 0.1 C (1C = 250 mA g^{-1}) in a hydrate-melt electrolyte during the second cycle.

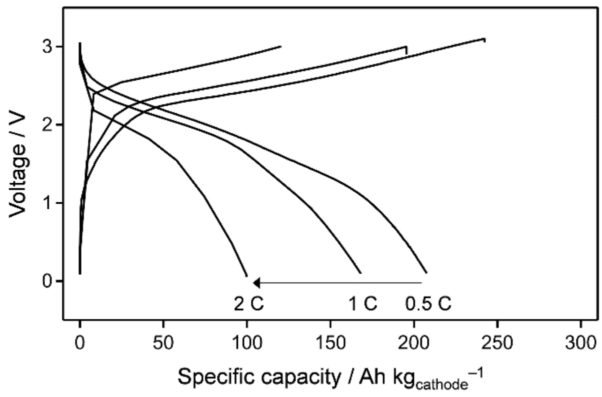

Figure S6. High-rate performance of the full cell of $Li_{1.2}Ni_{0.2}Mn_{0.6}O_2$ and $Li_4Ti_5O_{12}$ in $Li(TFSI)_{0.7}(BETI)_{0.3}\cdot 2H_2O$ electrolyte at 0.5, 1, and 2 C.
